# Effects of Light and Sound on the Prefrontal Cortex Activation and Emotional Function: A Functional Near-Infrared Spectroscopy Study

**DOI:** 10.3389/fnins.2017.00321

**Published:** 2017-06-09

**Authors:** Shota Hori, Koichi Mori, Takehisa Mashimo, Akitoshi Seiyama

**Affiliations:** ^1^Human Health Sciences, Graduate School of Medicine, Kyoto UniversityKyoto, Japan; ^2^Japan Society for the Promotion of ScienceTokyo, Japan; ^3^Department of Information and Media, Doshisha Women's College of Liberal ArtsKyoto, Japan; ^4^Media Design Department, Seian University of Art and DesignOtsu, Japan

**Keywords:** media art, neuroscience, sound, light, emotional change, prefrontal cortex, BCI

## Abstract

We constructed a near infrared spectroscopy-based real-time feedback system to estimate the subjects' emotional states using the changes in oxygenated hemoglobin concentration [Δ(oxy-Hb)] in the prefrontal cortex (PFC). Using this system, we investigated the influences of continual mild and equivocal stimuli consisting of lights and a reconstructed waterfall sound on Δ[oxy-Hb] in the PFC. The visual (light) and auditory (sound) stimuli changed randomly and independently, depending on the emotional states of the individual subjects. The emotional states induced by the stimuli were examined via a questionnaire rated on an 11-point scale, from +5 (pleasant) to −5 (unpleasant), through 0 (neutral), after the 5-min experiments. Results from 757 subjects revealed that Δ[oxy-Hb] in the PFC exhibited a weak, but significant, correlation with emotional change, with the given continual and mild stimuli similar to that experienced in response to the intense pleasant/unpleasant stimuli. Based on the results we discuss the generation of pleasant/unpleasant weak emotional change induced by mild and weak stimuli such as light and sound.

## Introduction

Recent advances in digital technology have made possible the interaction between humans (viewers) and media technology (such as computer graphics, digital video, robotics, networks, virtual reality, and the web), which is called interactive arts or media art (Edmonds et al., 2004; Edward, 2009). This type of art, which consists of static, dynamic-passive, dynamic-interactive, or dynamic-interactive (varying) systems (Edmonds et al., 2004) may be applicable to the development of an educational technology (Ariga and Mori, 2010) and a medical assisting technology, such as the brain-computer interface (BCI). Initially, the BCI techniques were developed to assist handicapped and paralyzed individuals to act independently and communicate successfully with others (Wolpaw et al., 2002; Dornhege et al., 2007). However, at present, patients require special training to be able to handle BCI (Blakely et al., 2014; Simon et al., 2015), which might prove nearly impossible for some of the disabled patients. Recently, near infrared spectroscopy (NIRS) has attracted attention for its use as a basic instrument for BCI techniques (Chaudhary et al., 2015; Von Lühmann et al., 2015; Shin et al., 2016), because of its moderate temporal resolution, noninvasive continuous measurement facility, compactness, and low interference with other techniques such as EEG and fMRI. In addition, NIRS is recently being applied for studies on emotional processing or the influence of emotions on cognition in the PFC (Bendall et al., 2016).

Hoshi et al. (2011) proposed the concept of Mind-Brain-Human Interface (MBHI) based on an emotional change-triggering training-free BCI model. The MBHI detects changes in emotions as neurophysiologic reactions within the patients and enables them to communicate with others (such as doctors, nurses, and aides) through the notifications based on their feelings. Hoshi et al. (2011) showed that intense pleasant/unpleasant emotional stimuli induce changes in cerebral blood flow (CBF) in the anterior prefrontal cortex (PFC), whereas intense pleasant emotional change induces decreases in CBF in the left dorsolateral PFC (l-DLPFC), and intense unpleasant emotional change induces increases in CBF in the bilateral ventrolateral PFC (b-VLPFC) detected via near infrared spectroscopy. These effects were confirmed recently using fMRI (Kohno et al., 2015). However, it is still unknown whether mild and/or equivocal stimulations induce such CBF changes in the PFC. Additionally, human brain activity patterns in response to the dynamics of continual emotional or feeling stimuli remain obscure.

In the present study, we hypothesized that functional near infrared spectroscopy (fNIRS) can quantitatively detect emotional changes caused by weak or mild affective stimuli, as well as by strong stimuli. Based on this assumption, a decrease in the fNIRS signal (i.e., oxy-Hb) of l-DLPFC represents an increase in pleasant emotions, while simultaneous increases in the oxy-Hb of b-VLPFC represent increases in unpleasant emotions. To test our hypothesis, we developed a NIRS-based real-time feedback system to measure the PFC blood flow change under continual mild and/or equivocal emotional light and sound stimuli to ascertain whether the PFC blood flow change is similar to that witnessed in response to intense emotional stimuli.

## Methods

### Participants

Seven hundred eighty-seven subjects participated in this experiment (294 men, 463 women, and 30 of unknown sex). The data obtained with the subjects who did not disclose their sex were excluded from analyses. Thus, data obtained with a total of 757 subjects was analyzed. The participants' age range was 6–78 years; mean: 32 +/− 14 (SD). All subjects provided their written informed consent prior to participating. This study was approved by the Kyoto University Graduate School and the faculty of the medical ethics committee and adhered to the tenets of the Declaration of Helsinki.

### fNIRS measurements of Δ[oxy-Hb] in the PFC

**Figure 1:**
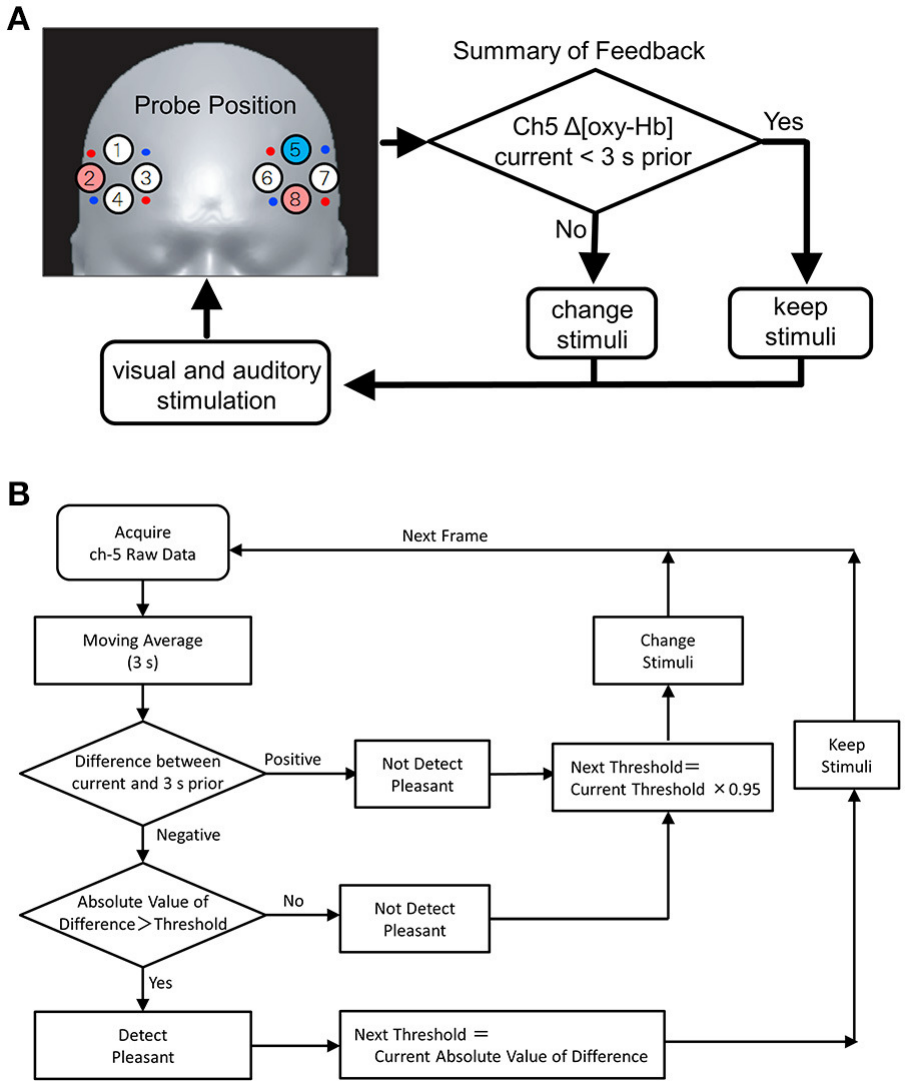
**(A)** NIRS probe position and feedback system. Red and blue circles on the left and right PFC denote the positions of incident and detection light guides. Circles with numbers are channel (Ch) positions. Changes in oxy-Hb at Ch 5 (blue) and Ch 8 (pink) in the left dorso- and ventrolateral PFC, as well as that at Ch 2 (pink) in the right ventrolateral PFC, were monitored. Signal change at Ch 5 was used as a feedback trigger, in which the stimuli were maintained as the values of oxy-Hb decreased, was lower than that of 3 s prior (yes); otherwise, the stimuli were changed randomly and independently. Feedback details are provided in the Methods. **(B)** Algorithm of the fNIRS base feedback system. Initially, the subject is exposed to arbitrary sound and color. Then, the fNIRS signal (oxy-Hb) at Ch 5 in the l-DLPFC is detected at every 50-ms interval. Subsequently, the signal processing as shown in the figure initiates and creates the next stimuli. The initial value of the threshold was set at 0, by modulating moving averaged value during 3 s prior measurement to be 0. The algorithm, Next Threshold = Current Threshold × 0.95, was used to the threshold gradually returns to the initial value.

The 19-channel multimodal fNIRS system, Foire-3000 (Shimadzu Co., Kyoto, Japan), was used for this study (Seiyama et al., 2012; Oonishi et al., 2014). The fNIRS system was used in the continuous wave mode, and three wavelengths, 780, 805, and 830 nm, were used to measure changes in the concentration of oxygenated [Δ[oxy-Hb)] and deoxygenated hemoglobin [Δ(deoxy-Hb)], according to the modified Beer-Lambert law. The source probe emitted light sequentially, in order to avoid cross-talk noise. For the present study, from among the 19 channels, we selected that which measured, and was positioned in, the area of left and right dorsolateral and ventrolateral PFC (Figure 1A). We tested the effects of the stimuli (detailed in the Methods) on the activation or deactivation of the PFC in 6 subjects (not included in the study) in separate experiments prior to the experimental measurements. Each channel is supposed to measure the changes at the mid-point between two probes and at a depth of 20–30 mm under the scalp. Optical probes (2 incident × 2 detection) of the fNIRS system were attached to the left and right lateral forehead with a distance of 3 cm between them. This alignment enabled us to measure 4 regions in both the lateral hemispheres, covering the Brodmann's areas 9 and 46 (dorsolateral region) and 45 (ventrolateral region). In our measurements, Channel (Ch) 5 was on the l-DLPFC, Ch 2 on the right-VLPFC (r-VLPFC), and Ch 8 on the left VLPFC (l-VLPFC). The measurements were performed at 70-ms sampling intervals.

### Feed-back system and signal processing

Figure 1A shows the concept of the feedback system used in the study and the PFC measurement area. The protocol of the feedback system is shown in Figure 1B. The fNIRS signal [i.e., Δ(oxy-Hb) at Ch 5], obtained at 70-ms sampling intervals, is transferred to an outer PC using a user datagram protocol. A 3-s moving average was used to create feedback signals of oxy-Hb for modifying the visual and/or auditory stimuli (Figure 1B, stimuli: described in the next section).

In separate experiments, conducted prior to the present study, we tested the feedback timing and effects of the present stimuli on the scalp blood flow and respiratory changes. using simultaneous measurements with fNIRS, Laser Doppler Flowmeter (FLO-C1, Omega Flow, Japan), and pulse oximeter (OLV-3100, Nihon Kohden) on 6 subjects. Additionally, we also determined the suitable sampling interval time in the range from 25 to 100 ms and moving average time from 1 to 5 s. Shorter sampling interval and moving average times caused nosy signals. In contrast, longer sampling interval and moving average time possibly include both, cognitive functions of the brain and emotional change (Hoshi et al., 2011), and thus resulted in the loss of real-time responses to changes in the stimuli. Thus, the prementioned sampling time and moving average time were selected for the measurement systems of the study.

### Visual and auditory stimuli

**Figure 2:**
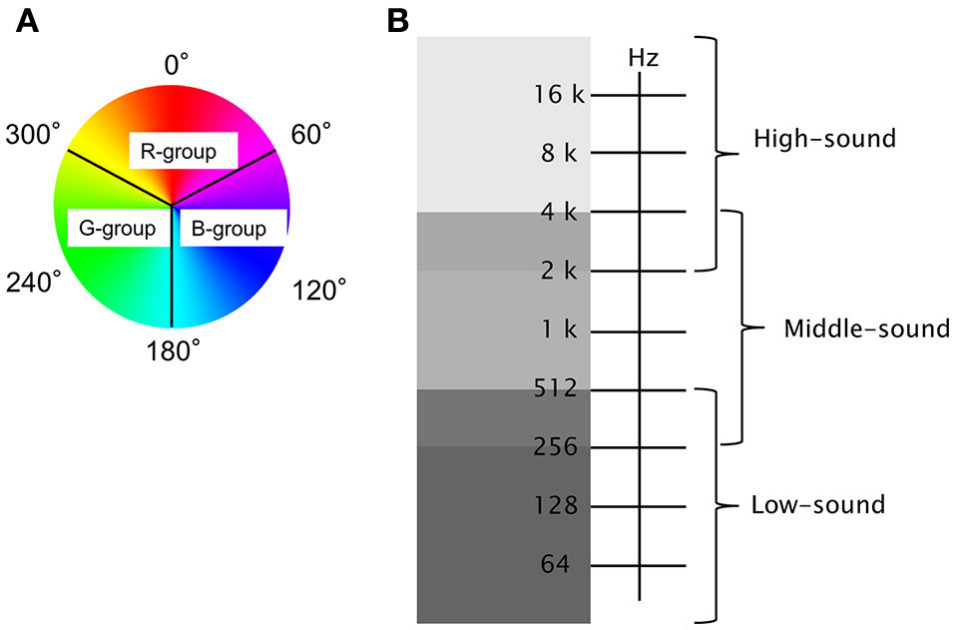
Analyses of light and sound stimuli: The R, G, and B color groups in HSB and sound frequency components divided into three groups (High, Middle, and Low) were classified in **(A,B)**, respectively.

The illumination light consisted of 3 types of LED lights: red (R), green (G), and blue (B) (Mega Pixel LED, American DJ). The illuminations of the individual LEDs were regulated by a feedback PC to ensure a gradation sequence between 0 and 100. Then, the color (i.e., R, G, and B) groups were calculated in accordance with hue, saturation, and brightness (HSB) color space (Figure 2A). The light generated with the LED system was projected on the white surface of a cylindrical tube screen (540 cm in length and 180 cm in diameter), which caused the subjects to experience a whole-body illumination. Using the feedback PC, a waterfall sound was divided into 10 band frequencies, as follows: f(Hz) ≥ 16,000, 16,000 > f ≥ 8,000, 8,000 > f ≥ 4,000, 4,000 > f ≥ 2,000, 2,000 > f ≥ 1,000, 1,000 > f ≥ 512, 512 > f ≥ 256, 256 > f ≥ 128, 128 > f ≥ 64, and f ≥ 64 Hz (Figure 2B). The divided sounds were then equalized and reconstructed as stimuli, presented through headphones. The stimuli were changed randomly according to a feedback loop (see also Figure 1B and its legend). The feedback loop consisted of the following steps: (1) After entering the tube stage, the subject was given an arbitrary color and sound as the initial stimuli; (2) When an increase in Δ[oxy-Hb] was detected at Ch 5, the color and sound stimuli were randomly and independently given to the subject. In contrast, when a decrease in Δ[oxy-Hb] was detected at Ch 5, the color and sound stimuli were continuously given to the subject until oxy-Hb increased. The judgment of an increase or decrease in Δ[oxy-Hb] at Ch 5 as the feedback signal was performed by comparing it with the value of oxy-Hb 3 s prior.

### Questionnaire

After the experiment, the participants answered a questionnaire. The emotional states induced by the stimuli were examined on an 11-point scale, from +5 (pleasant) to −5 (unpleasant), through 0 (neutral) after the 5-min experiments. We then compared the valency of this questionnaire with the rates of increase in oxy-Hb during the 5-min experiments, for individual participants. We obtained answers to the questionnaire from 298 of the 757 participants [118 men and 181 women, with an age range of 6–78 years; M = 32 +/− 14 (SD)].

### Statistical analyses

Data are shown as the mean plus or minus standard deviation (S.D.). The statistical significance (the *P*-value) was evaluated using a multiple comparison test, Mann-Whitney *U*-test with Bonferroni correction test. Significance was taken at *P* < 0.05. Correlation coefficient between Ch 5 and Chs 2 and 8 of fNIRS signals was obtained with the Pearson's correlation coefficient “r,” which was converted to a Zr-value in accordance with Fisher's Z-transformation expression Zr = (1/2)^*^ln{(1 + r)/(1-r)} for statistical analyses.

## Results

### Measurements of stimuli-induced changes in cerebral blood flow in the PFC

Experiments were performed with 757 subjects. Δ[oxy-Hb]s at Ch 5 and Ch 8 in the left dorso- and ventrolateral PFC, and that at Ch 2 in the right ventrolateral PFC were monitored.

**Figure 3:**
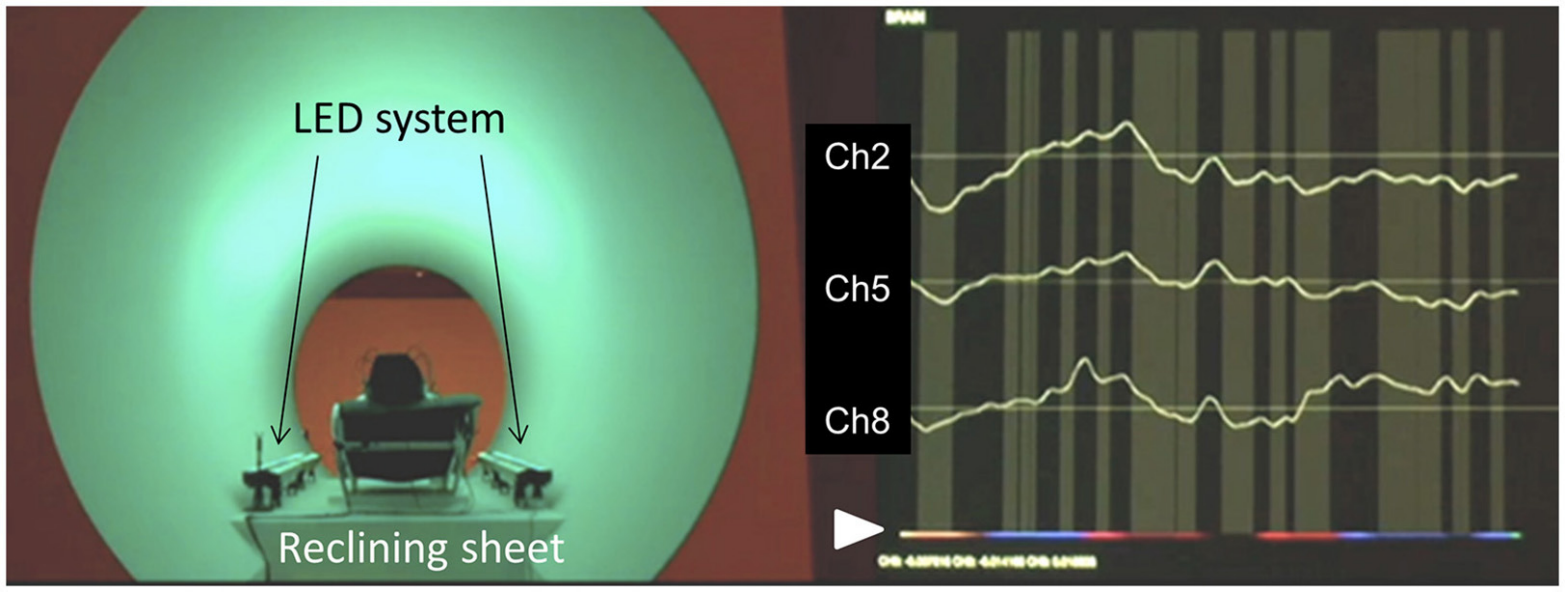
Example of the experiments: Left, subject reclines in the cylindrical tube stage (540 cm in length and 180 cm in diameter), and is equipped with the NIRS probes and headphones. The LED system provided a whole-body illuminating light source to the cylindrical surface, and the reconstructed waterfall sound was administered through the headphones. Right, changes in oxy-Hb at Ch 2, Ch 5, and Ch 8 were continuously monitored every 3 s. The arrowhead at the bottom shows the stimulus light generated by the LED system through the feedback algorithm. The vertical gray streak denotes decreases in oxy-Hb at Ch 5. Actual changes in signals and stimuli are shown in the Supplemental Video file.

In the present study, according to the results of Hoshi et al. (2011), we adopted an assumption that decrease in Δ[oxy-Hb] at Ch 5 (in the area of the l-DLPFC) reflects an increase of pleasant emotions, whereas increases in Δ[oxy-Hb] at both, Chs 2 and 8 (i.e., in the areas of the right- and left-VLPFC) reflect increase of the unpleasant emotions. Thereafter, the signal change at Ch 5 was used as a feedback trigger (see Figure 1), where the stimuli were maintained at the value of oxy-Hb lower than that of 3 s prior (“yes” assuming the pleasant state), whereas the stimuli were changed randomly and independently (“no” assuming the unpleasant state). Figure 3 shows a typical example of stimuli-induced Δ[oxy-Hb] at Chs 2, 5, and 8. The arrowhead at the bottom shows the stimulus light generated by the LED system through the feedback algorithm (detailed in the Methods section). The vertical gray streak denotes decreases in Δ[oxy-Hb] at Ch 5. Actual Δ[oxy-Hb]s at Chs 2, 5, and 8 accompanying the stimuli are shown in the supplemental video file.

**Figure 4:**
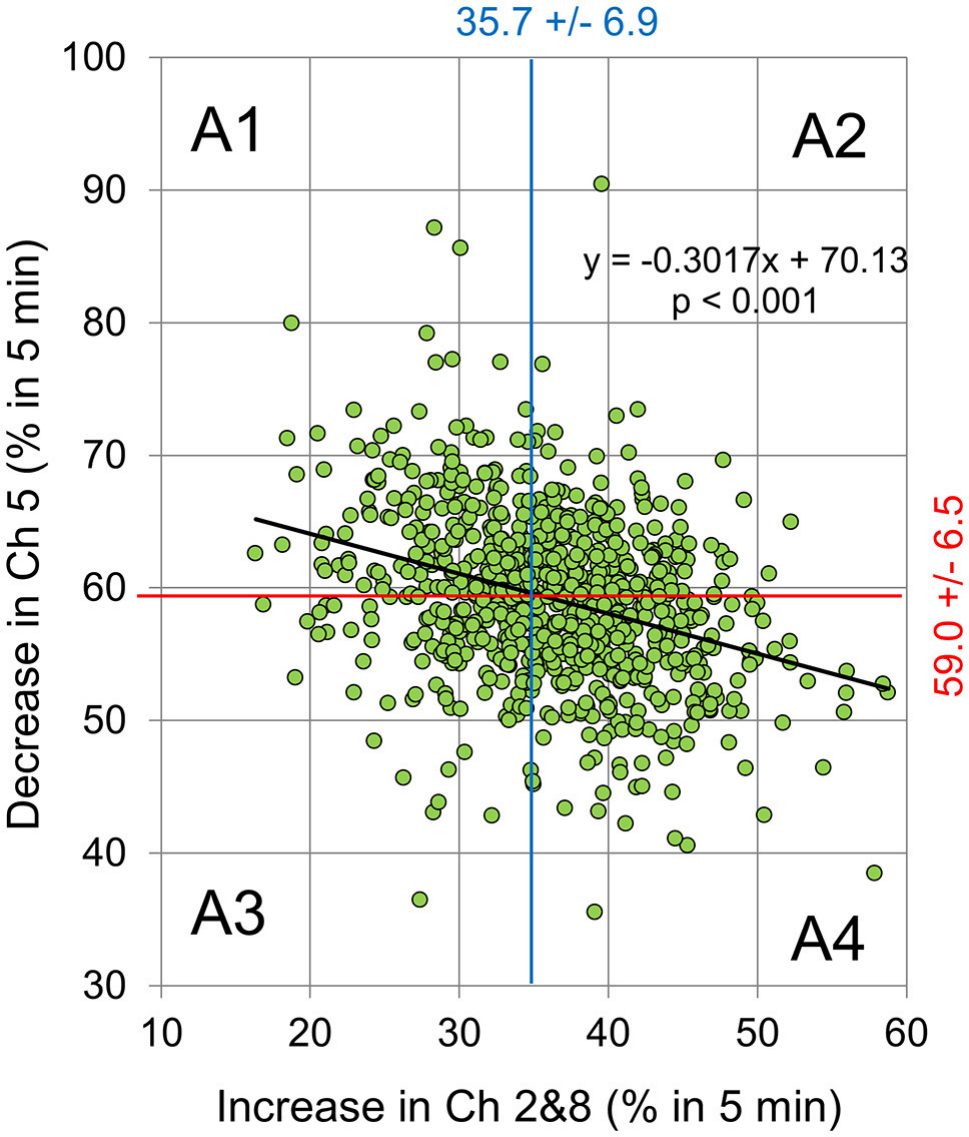
The relationship between decreased Ch 5 signal and simultaneously increased Ch 2 and 8 signals during the 5-min experiment for individual subjects. The changes were expressed as % of decreased (or increased) duration for 5 min (i.e., a decrease or increase for 5 min denotes 100%). There was a weak, but significant, correlation between the decrease at Ch 5 and the increase at Chs 2 and 8 (*Y* = −0.3017 ^*^ *X* + 70.13, *R*^2^ = 0.1041, *p* < 0.001). The vertical blue and horizontal red lines represent mean values of the changes in the increased Ch 2 and 8 signals (35.7% +/− 6.9 S.D.), as well as those of the decreased Ch 5 (59.0% +/− 6.5 S.D.). Here, the areas divided by these lines were classified as: Area 1 (A1: *Y* > 59.0 and *X* < 35.7%); Area 2 (A2: *Y* > 59.0 and X > 35.7%); Area 3 (A3: *Y* < 59.0and *X* < 35.7%); and Area 4 (A4: *Y* < 59.0 and *X* > 35.7%).

Figure 3 shows the relationship between the decreased Δ[oxy-Hb] at Ch 5 and simultaneously decreased Δ[oxy-Hb] at Chs 2 and 8 during a 5-min experiment for all 757 subjects individually. The changes were expressed as a percentage of a decreased (or increased) duration of 5 min. We observed weak, but significant, negative correlations between the decrease at Ch 5 and the increase at Chs 2 and 8 (*p* < 0.001) (Figure 4).

### Questionnaire of stimuli-induced emotional changes

Emotional states induced by the stimuli were examined using a questionnaire assessing items on an 11-point scale, from +5 (pleasant) to −5 (unpleasant), through 0 (neutral), after the 5-min experiments. We successfully obtained answers from 298 of the 757 participants. The subjects were divided into 4 groups according to their NIRS signal changes (see also Figure 4), and the questionnaire responses were analyzed in accordance with their classifications. Table 1 shows the averaged questionnaire responses for each group. Although there were no statistical differences, the results, featuring a pattern of A1 > A2 = A3 > A4, represented the changes in the NIRS signals well, i.e., A1 group showed relatively high Ch 5 signal change and the A4 group showed relatively high Ch 2- and 8-signals changes.

**Table 1:** Averaged scores of emotional level [pleasant (+5)—unpleasant (−5)] during the 5-min experiment.

**Group**	**A1**	**A2**	**A3**	**A4**
Mean	2.54	2.48	2.48	2.29
S.D	2.21	2.25	2.15	2.12
N	87	60	60	91

*Subjects were divided into 4 groups based on NIRS signal change (see Figure 4 and its legend). Means and standard deviation (S.D.s) are shown. “N” denotes the number of subjects in the classified area*.

Table 2 shows the effects of color and sound frequency on the subjects' emotional states. The combinations of the color and sound frequency components during the decrease in Δ[oxy-Hb] at Ch 5 (the pleasant state) and the increase in Δ[oxy-Hb]s at Chs 2 and 8 (the unpleasant state) are shown in top and bottom scales, respectively. With both emotions, the color components (R, G, or B) produced similar effects. For example, in the case of pleasant emotions, the effects of the R, G, and B colors were 33.5, 33.6, and 32.9%, respectively. In the case of unpleasant emotions, their effects were 34.4, 32.8, and 32.8%, respectively. In contrast, the effect of sound on the emotional changes was remarkable. In both the cases (i.e., the pleasant and unpleasant emotions), high-frequency sound elicited emotional change in about 43% of the emotional states (43.8% in the pleasant and 43.3% in the unpleasant, respectively). Low-frequency sounds were also effective in eliciting emotional changes (~32% in both cases), but this effect was weaker than that of the high-frequency sounds.

**Table 2:** Effects of color (row) and sound frequency (column) on emotional state.

	**R%**	**G%**	**B%**	**Total%**
**PLEASANT (%)**				
L	10.0	10.9	10.6	31.5
M	8.1	8.5	8.1	24.7
H	15.4	14.2	14.2	43.8
Total	33.5	33.6	32.9	100
**UNPLEASANT (%)**				
L	10.5	10.7	10.6	31.8
M	8.2	8.7	8.1	25.0
H	15.7	13.5	14.1	43.3
Total	34.4	32.8	32.8	100

*Top: Combinations of color components and sound frequencies during the decrease of Ch 5 fNIRS signals (indicating pleasant state). Bottom: Combinations of color components and sound frequencies during the increase of Chs 2 and 8 fNIRS signals (indicating unpleasant state). The color range of R, B, and G in the HSB color space and the frequency range of the equalized and reconstructed sound (i.e., H, M, and L) are shown in Figures 2A,B, respectively. Each number denotes the fractional contribution to each emotion (pleasant: table at the top and unpleasant: at the bottom)*.

## Discussion

Our aim was to investigate whether fNIRS can detect emotional changes caused by weak or mild affective stimuli. We used the various colors produced by a combination of LED lights and the equalized sounds of a waterfall, as simultaneous visual and auditory stimuli for this experiment. The emotional changes caused by the stimuli were assessed using a questionnaire, and then these results were compared with the changes in oxy-Hb in the bilateral PFC. We obtained a correlation between fNIRS signal changes and questionnaire responses.

Here, we discuss the detection of mild or weak emotional changes using fNIRS and the possibility of constructing an emotion based BCI—i.e., MBHI—based on an emotional change-triggering training-free BCI model.

### Detection of weak emotional change with fNIRS

The key role of the lateral PFC (LPFC) in higher-order cognitive functions is well-established, as is its role in the self-regulation of emotion (Beauregard et al., 2001; L'evesque et al., 2003). Using strong affective visual stimuli and fNIRS, several authors have recently detected emotional or affective change in the LPFC of human subjects (Marumo et al., 2009; Hoshi et al., 2011). Hoshi et al. (2011) used near infrared spectroscopy to detect decreases in CBF in the l-DLPFC in response to intense pleasant emotional change, as well as increases in CBF in the b-VLPFC in response to intense unpleasant emotional change. In the present study, we adopted Ch 5 for the measurements in the cortical area of the l-DLPFC, and Chs 2 and 8 for the b-VLPFC fNIRS measurements (see Figure 1). Additionally, as described earlier, we assumed that a decrease in Δ[oxy-Hb] at Ch 5 reflects increase of pleasant emotions, whereas increases in Δ[oxy-Hb] at both, Chs 2 and 8, reflect an increase of unpleasant emotions. Under the present experimental conditions, we observed weak, but significant, correlations between the decrease at Ch 5 and increases at Ch 2 and 8 signals (Figure 4, *p* < 0.001). Furthermore, the questionnaire results concerning emotional change occurring during our experiment (Table 1) coincided well with the fNIRS results (see also Figure 4) and supported the validity of our assumption.

It should be noted that several recent reports have cautioned that the skin (scalp) blood flow in the frontal cortex considerably influences fNIRS signals especially during active-stimuli tasks such as, semantic continuous performance task including a verbal fluency task and N-back task (Takahashi et al., 2011, 2016; Kirilina et al., 2012). In the present study, we used passive-stimuli tasks, where subjects were made to listen to sounds and observe lights, without thinking and or performing an action and tested whether the passive-stimuli elicit the scalp blood flow. We did not observe scalp blood flow in separate preliminary experiments with 6 subjects. In addition, no significant changes were detected in the pulse rate measured at the fingertip.

These results suggest that (1) mild or weak stimuli, like lights and sounds, elicit weak pleasant and/or unpleasant emotion-associated changes in the DLPFC and (2) weak pleasant and/or unpleasant emotions can be detected at the l-DLPFC and b-VLPFC, respectively.

### Effects of stimuli (lights and sounds) on emotional change

It is reported that the reddish color elicits negative feeling (Elliot and Maier, 2014) whereas blue and green colors have the most pleasant hues (Valdez and Mehrabian, 1994). However, in the present study, we did not observe differences with respect to the reddish, blueish, and greenish colors on the pleasant or unpleasant emotions (see Figure 2A, Table 2). Effects of visual stimuli on auditory perception or auditory stimuli on visual perception are well-known (e.g., Burr and Alais, 2006). Thus, the combined effect of light and sound stimuli may have affected the preference for colors under the present experimental condition. However, the sound effects were in the order of H > L > M in both, the pleasant and unpleasant conditions (see Figure 2B). The frequency range of M (from 4,000 to 256 Hz) is similar to that experienced in our daily lives. Thus, there is a possibility that the M frequency range of sound stimuli did not cause or caused low emotional change (pleasant or unpleasant depending on the individual) in the subjects, whereas the H or L frequency sounds may elicit emotional change due to their unusual features. Plichta et al. (2011) reported that the pleasant and unpleasant auditory stimuli evoke activation of the auditory cortex and showed similar response in fNIRS measurements. Although the stimuli were different from those of previous studies, these results suggest that positive connections between the auditory cortex and the VLPFC are induced by unpleasant emotions whereas negative connections between the auditory cortex and DLPFC are induced by pleasant emotions.

### Biofeedback system for mind-brain-human interface

Another aim of this study was to construct an emotion based BCI—i.e., MBHI—based on an emotional change-triggering training-free BCI model. In the present study, we used Δ[oxy-Hb] at Ch 5 as a feedback signal to detect and continuously maintain a “pleasant” emotional state. The results in Figure 4 show that our assumption that decreases in Δ[oxy-Hb] at Ch 5 reflects pleasant state, and the satisfactory functioning of feedback system was confirmed. It should be noted that the change (i.e., increase or decrease) in Δ[oxy-Hb] at Ch 5 in l-DLPFC was almost similar to those at Chs 2 and 8 in both sides of the VLPFC. This feature also possibly supported our assumption that the increase in Δ[oxy-Hb] at both Chs 2 and 8 reflects pleasant state (i.e., in turn decrease in Δ[oxy-Hb] at both Chs 2 and 8 reflects unpleasant state). As shown in Figure 3 and the supplemental video, Δ[oxy-Hb] changed frequently; this phenomena may reflect the processes of acclimation or boredom to the stimuli and seeking of new, pleasant stimuli, with a combination of sound and light, by the subjects. Finally, it should be noted that in the separate experiments on 6 subjects conducted prior to the present study, we also tested a possibility that increases in Δ[oxy-Hb] at both Chs 2 and 8 function as feedback signals to detect and continuously maintain “unpleasant” emotional state. However, several subjects stated experiencing discomfort during the test, thus this experiment was not ethically accepted.

## Conclusions

In this study, we have shown that mild or weak visual and auditory stimuli elicit both pleasant and unpleasant emotional changes in the LPFC, and these emotional changes can be detected by a real-time feedback fNIRS system. Although further refinement of this system and experiments are required, the present results suggest the practicability of an emotional change-triggering training-free BCI model, the MBHI.

## Ethics statement

This study was carried out in accordance with the recommendations of the Kyoto University Graduate School and the faculty of the medical ethics committee with written informed consent from all subjects. All subjects gave written informed consent in accordance with the Declaration of Helsinki. The protocol was approved by the Kyoto University Graduate School and the faculty of the medical ethics committee.

## Author contributions

SH analyzed results and wrote this paper, KM and TM designed and performed the experiments, discussed the results, and reviewed the manuscript; AS discussed the results and wrote this the paper. KM and AS worked together to obtain funding.

### Conflict of interest statement

The authors declare that the research was conducted in the absence of any commercial or financial relationships that could be construed as a potential conflict of interest.
